# *In-Silico* Analyses of Sesquiterpene-Related Compounds on Selected *Leishmania* Enzyme-Based Targets

**DOI:** 10.3390/molecules19055550

**Published:** 2014-04-29

**Authors:** Freddy A. Bernal, Ericsson Coy-Barrera

**Affiliations:** Laboratorio de Química Bioorgánica, Departamento de Química, Facultad de Ciencias Básicas y Aplicadas, Universidad Militar Nueva Granada, Cundinamarca 250240, AA 49300, Colombia; E-Mail: freddy.bernal@unimilitar.edu.co

**Keywords:** *Leishmania*, *in-Silico*, molecular docking, sesquiterpene

## Abstract

A great number of sesquiterpenes are reported in the available literature as good antileishmanial leads. However, their mode of action at the molecular level has not been elucidated. The lack of molecular studies could be considered an impediment for studies seeking to improve sesquiterpene-based drug design. The present *in silico* study allows us to make important observations about the molecular details of the binding modes of a set of antileishmanial sesquiterpenes against four drug-enzyme targets [pteridine reductase-1 (PTR1), *N*-myristoyl transferase (NMT), cysteine synthase (CS), trypanothione synthetase (TryS)]. Through molecular docking it was found that two sesquiterpene coumarins are promising leads for the PTR1 and TryS inhibition purposes, and some xanthanolides also exhibited better affinity towards PTR1 and CS binding. In addition, the affinity values were clustered by Principal Component Analysis and drug-like properties were analyzed for the strongest-docking sesquiterpenes. The results are an excellent starting point for future studies of structural optimization of this kind of compounds.

## 1. Introduction

Parasites of the genus *Leishmania* are trypanosomatid protozoa that cause the neglected disease known as leishmaniasis, which infects some 15 million people around the world in three clinical forms: cutaneous, mucocutaneous and visceral [1,2]. Its emergence as an opportunistic pathogen has generated a public health interest and an ongoing need to control it. However, its treatment still remains based on the use of pentavalent antimony salts as first-line drugs, or the use of amphotericin B and pentamidine as second-line drugs, which are often toxic, some have an unknown mode of action, and are usually marginally effective, plus the added problem that there are outbreaks of resistance [3,4]. So far, there are not enough advances in the replacement of these drugs, with some cases of uncertainly effective therapy, although there are some clinical trials based on known drugs such as allopurinol (a protein synthesis inhibitor), AmBisome^®^ (a formulation of amphotericin B in liposomes) and ketoconazole (an inhibitor of sterol synthesis) [5].

Several metabolic pathways are currently under study in order to identify critical enzymes for inhibition purposes with the aim of exploiting them for the treatment of parasitic diseases. Some of the most interesting enzymes are those involved in metabolism of glucose, sterols, fatty acids and nucleotides and those key enzymes for protein biosynthesis and for the maintenance of trypanothione and polyamine levels [6].

A highly valuable reaction to protect and maintain vitally important intracellular amounts of tetrahydropterin—which has been proven as an essential part of the growth—is the reduction of conjugated and non-conjugated pterins, such as reduced biopterin to dihydrobiopterin, which is catalyzed by pteridine reductase (PTR1) [7,8]. Because PTR1 is less sensitive to the effect of methotrexate, but also catalyzes the reduction of folates, it explains the therapeutic failure of these drugs against trypanosomatid parasites [7].

It is well-known that *Leishmania* requires cysteine for protein biosynthesis and as a precursor of trypanothione, with an essential role in redox metabolism and antioxidant defense [9]. A *de novo* route to produce cysteine, selectively present in some microorganisms (but absent in mammals), is catalyzed by cysteine synthase (CS), in a two-stage process from serine [10]. In addition, the bifunctional trypanothione synthetase-amidase catalyzes biosynthesis and hydrolysis of the trypanothione, a glutathione-spermidine conjugate, which is critical for intracellular thiol-redox unique to trypanosomatids. The synthetase *C*-terminal domain displays the ATP-grasp fold, which binds nucleotide in a well-defined fashion [11].

Some studies have identified *N*-myristoyltransferase (NMT) as an appropriate drug target against protozoan parasitic diseases. NMT is ubiquitous in eukaryotic cells catalyzing the addition of the myristate to the *N*-terminal glycine residue of some proteins [12]. Protein *N*-myristoylation is important for targeting proteins to membrane sites, mediating protein–protein interactions and stabilizing protein structures [13].

Several sesquiterpene-related compounds isolated from natural sources have shown *in vitro* effectivity against promastigotes and amastigotes of *Leishmania* and *in vivo* activity [14,15]. However, their mode of action has not been elucidated. Recently, a work reported an *in silico* study through molecular docking of a large number of terpenoids within the active site of 29 enzymes from *L. major*, *L. donovani*, *L. mexicana* and *L. infantum* [16]. In this study, *ca.* 100 different sesquiterpenoids (mainly sesquiterpene lactones) were also docked, with the results indicating target selectivity, e.g., germacranolide sesquiterpenoids exhibited selectivity to *L. major* methionyl *t*-RNA synthetase (*Lm*MRS) and dihydroorotate dehydrogenase (*Lm*DHODH). Among the targeted enzymes, *Lm*PTR1 and *Ld*NMT were also docked with test sesquiterpenes with reasonable affinity. However, a number of bioactive sesquiterpenoids were not included in that study and *Lm*CS and *Lm*TryS enzymes are not targeted. Therefore, as part of our interest in finding new prototypes against leishmaniasis, in this work a study through molecular docking and residual interactions of the molecular basis of action at the *in silico* level of 123 sesquiterpene-related compounds (possessing antiparasitic activity) within the active site of *Lm*PTR1, *Ld*NMT, *Lm*CS, and *Lm*TryS, is described. These analyses showed very important details at the molecular level that should prove useful for the development of therapeutics from sesquiterpene-based prototypes.

## 2. Results and Discussion

### 2.1. Molecular Docking of Sesquiterpenes

**Figure 1:**
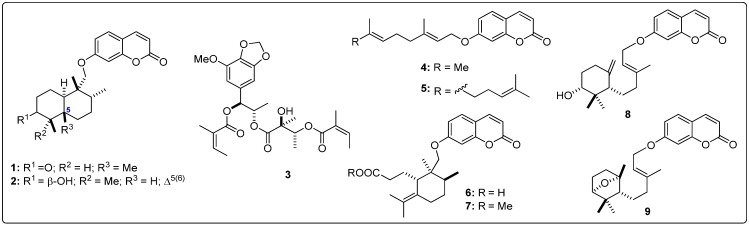
Structures of the tested sesquiterpene coumarins [18].

**Figure 2:**
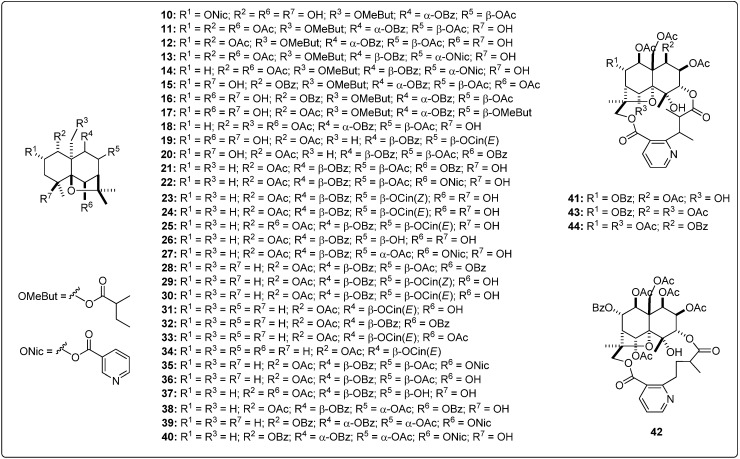
Structures of the tested agarofuran sesquiterpenes [19,20,21,22,23].

**Figure 3:**
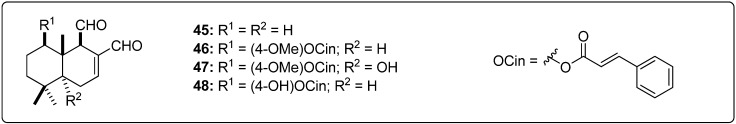
Structures of the tested drimane sesquiterpenes [24].

**Figure 4:**
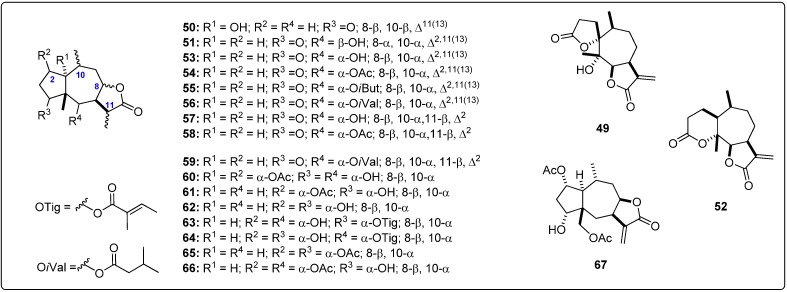
Structures of the tested pseudoguaianolide sesquiterpenes [25,26,27].

**Figure 5:**
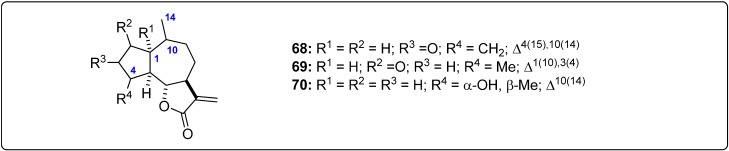
Structures of the tested guaianolide sesquiterpenes [26,28,29].

**Figure 6:**
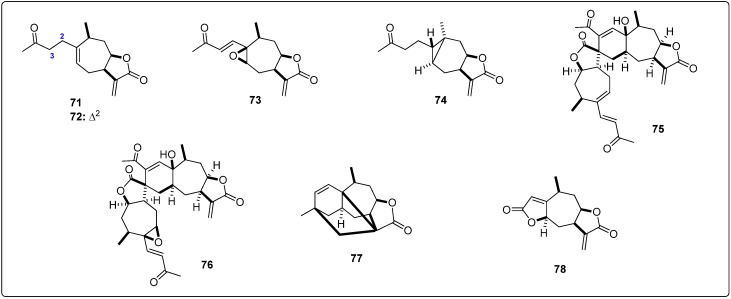
Structures of the tested xanthanolide sesquiterpenes [27].

**Figure 7:**
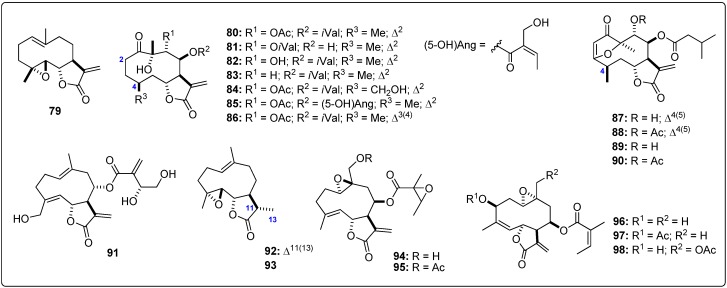
Structures of the tested germacranolide sesquiterpenes [27,30,31,32].

**Figure 8:**
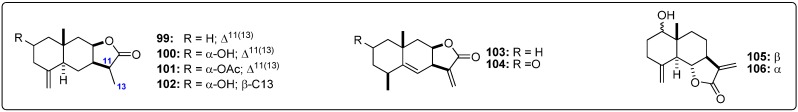
Structures of the tested eudesmanolide sesquiterpenes [27].

**Figure 9:**
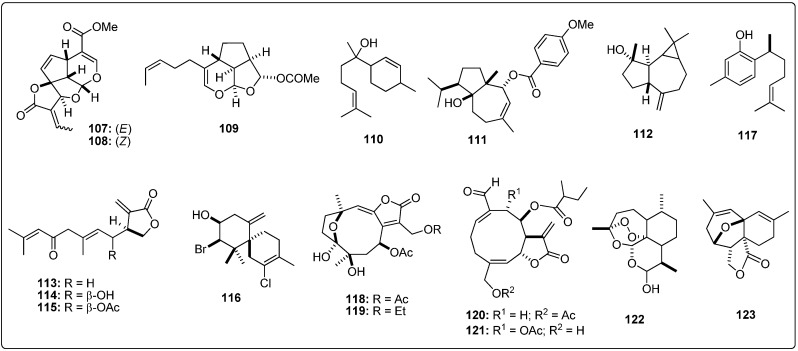
Structures of the tested miscellaneous sesquiterpenes [24,33,34,35,36,37,38,39,40,41,42].

Structural information about the test sesquiterpenoids were acquired from different published works reporting antileishmanial activity of natural-occurring sesquiterpenes. The IUPAC and common names can be found in the Supplementary Material. The compounds were further subdivided by common moieties such as sesquiterpene-coumarins, agarofurans, drimanes, pseudoguaianolides, guaianolides, xanthanolides, germacranolides, eudesmanolides and miscellaneous, whose structures are shown in Figure 1, Figure 2, Figure 3, Figure 4, Figure 5, Figure 6, Figure 7, Figure 8 and Figure 9. Each ligand structure was separately submitted to a conformational search using MMFF and the geometry of the most stable conformer was then optimized by semi-empirical AM1 parametrization. The most stable conformer of each optimized structure was imported into Autodock/Vina to be docked into the active site of *Lm*PTR1, *Ld*NMT, *Lm*CS and *Lm*TryS enzymes. The structural data of the enzymes were imported from the Protein Data Bank (PDB) (Table 1).

**Table 1:** Data of the test enzymes.

Enzyme	PDB ID *^a^*	Resolution [Å]	Source	Abbreviation	Reference *^b^*
Pteridine reductase	2QHX	2.61	*L. major*	*Lm*PTR1	[17]
Cysteine synthase	4AIR	1.80	*L. major*	*Ld*NMT	[10]
*N*-Myristoyl transferase	2WUU	1.42	*L. donovani*	*Lm*CS	[13]
Trypanothione synthetase	2VOB	2.30	*L. major*	*Lm*TryS	[11]

*^a^* Assigned code in the Protein Data Bank (PDB); *^b^* Reference including the active site.

The resulting docking energies (vina scores as affinity values in kcal/mol) of sesquiterpenoids are summarized in Table 2. Szowitsiacoumarin B (**2**) was found to be the strongest-docking sesquiterpene coumarin. The sesquiterpene coumarins exhibited overall stronger docking energies towards the *Leishmania* protein targets. This fact is observed by the mean value of the docking energies for each sesquiterpene-type skeletal (Table 3). The RSD percentages were found to be in a 5.1%–11.4% range indicating dispersion between the affinities for each type of sesquiterpenes which might be correlated with enzyme selectivity. In contrast, pseudoguaianolides, guaianolides, and germacranolides revealed the highest-docking energies and lowest-RSD percentages. Agarofurans **48**–**50**, **55**, **90**–**91** also exhibited stronger docking energies, although they showed selectivity towards PTR1 and NMT.

**Figure 10:**
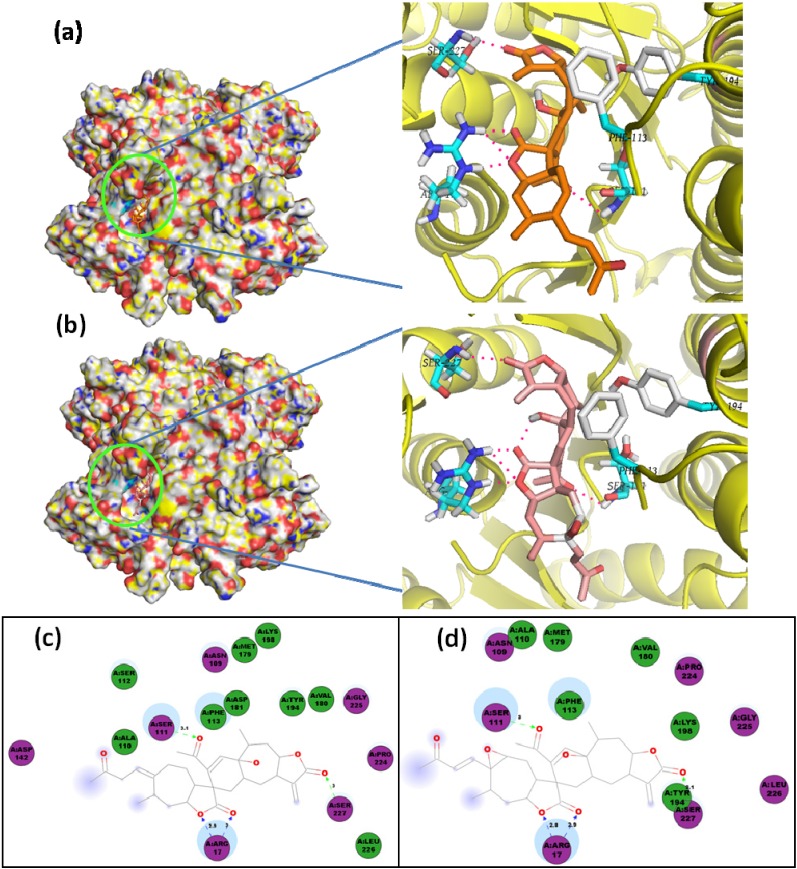
Lowest-energy docked pose with *Lm*PTR1 of: (**a**) sesquiterpene **75**; (**b**) sesquiterpene **76**; 2D representation of protein ligand interactions of: (**c**) compound **75**; (**d**) compound **76**.

In the case of the docking with PTR1, dimeric xanthanolide compounds **75**–**76** exhibited the best affinity values (‒10.6 kcal/mol), with even lower values than that of the control used, DB07765 (‒10.2 kcal/mol). Their lowest-energy docked poses are shown in Figure 10. Both complexes share identical *H*-bond interactions with Ser111, Ser227 and Arg17, being the crucial contacts for the ligand-enzyme docking. In addition, as shown in the 2D representation of protein ligand interactions (Figure 10c), a receptor contact through Phe113 contributes to the non-polar stabilization in the hydrophobic guaianolide moiety. These two compounds were reported by Schmidt *et al.* [27] with reasonable antileishmanial activity against *L. donovani* amastigotes with IC_50_ values in the 22–27 µM range. Our results indicate that compounds **75**–**76** could serve as leads for further structural optimization studies towards PTR1 inhibition.

In addition, compounds **75**–**76** share as structural feature an α-methylene γ-lactone moiety. This moiety in sesquiterpene lactones (STLs) is considered to be capable for reacting in nucleophilic Michael additions (acting as Michael acceptor) with sulfhydryl-(-SH) or amino-(-NH_2_)-containing residues, such as cysteine, lysine and histidine [43,44]. Several studies have indicated that the formation of this type of Michael adducts is correlated with the biological activity of STLs [45]. Analyzing the 2D representation of protein ligand interactions (Figure 10c–d) for lowest-energy docking pose of **75**–**76**, and other test α-methylene-containing STLs (data not shown), Lys198 was found to be the only residue within the *Lm*PTR1 binding pocket that satisfies the structural requirement for a possible Michael addition, but due to the *H*-bond interaction (2.1 Å) between the lactone carbonyl group and Ser222, the Lys198 residue is placed at the other face of the molecule, which eventually might hinder the formation of a Michael adduct.

**Table 2:** Docking energies *^a^* (kcal/mol) of sesquiterpenoids with *Leishmania* enzyme targets.

No	*Lm*PTR1	*Lm*CS	*Ld*NMT	*Lm*TryS	No	*Lm*PTR1	*Lm*CS	*Ld*NMT	*Lm*TryS	No	*Lm*PTR1	*Lm*CS	*Ld*NMT	*Lm*TryS
**sesquiterpene coumarins**					**drimanes**					**germacranolides *cont.***				
**1**	−9.9	−11.2	−6.6	**−8.9**	**45**	−6.9	−7.6	−4.5	−7.0	**86**	−8.0	−9.0	−6.2	−8.3
**2**	**−10.0**	**−11.8**	**−6.8**	−8.5	**46**	−8.9	−9.9	−5.9	−7.9	**87**	−8.1	−9.5	−5.5	−7.7
**3**	−7.8	−10.0	−5.6	−7.4	**47**	−8.6	−9.9	−6.1	−8.3	**88**	−8.1	−8.7	−5.2	−7.5
**4**	−7.4	−9.5	−5.1	−7.9	**48**	−8.9	−10.8	−5.5	−7.8	**89**	−8.4	−9.0	−5.7	**−8.7**
**5**	−7.5	−10.0	−4.9	−8.2	**pseudoguaianolides**					**90**	−8.0	−9.6	−5.7	−7.9
**6**	−9.5	−10.2	−6.4	−8.4	**49**	−8.6	−8.5	−5.1	−7.9	**91**	−8.2	−9.0	−6.3	−7.4
**7**	−8.9	−10.2	−6.3	−8.4	**50**	−7.9	−8.5	−5.2	−7.0	**92**	−7.6	−8.7	−5.8	−7.4
**8**	−9.2	−10.2	−6.0	−8.1	**51**	−8.1	−9.6	−5.6	−8.6	**93**	−7.7	−8.9	−5.8	−7.3
**9**	−8.6	−10.7	−6.2	−8.2	**52**	−8.2	−10.5	−5.7	−7.9	**94**	−8.1	−8.6	−5.7	−7.7
**agarofurans**					**53**	−8.0	−8.5	−5.5	−7.3	**95**	−7.7	−8.7	−5.9	−7.5
**10**	−8.8	−8.5	−5.5	−7.0	**54**	−8.3	−9.2	−6.1	−7.6	**96**	−8.6	−8.7	−5.7	−7.1
**11**	−7.5	−7.4	−4.7	−6.6	**55**	−8.3	−8.9	−5.4	−7.6	**97**	−8.8	−9.2	−5.5	−7.3
**12**	−7.8	−7.6	−5.1	−6.5	**56**	−8.2	−9.7	−5.7	−8.0	**98**	−8.3	−9.0	−5.1	−7.2
**13**	−7.4	−8.9	−5.1	−3.2	**57**	−7.8	−9.1	−5.7	−7.3	**eudesmanolides**				
**14**	−7.2	−9.0	−5.2	−3.1	**58**	−8.1	−8.5	−5.9	−7.5	**99**	−7.8	−8.4	−5.1	−7.1
**15**	−8.2	−8.1	−5.0	−5.6	**59**	−8.3	−9.4	−5.9	−8.0	**100**	−7.8	−8.4	−5.1	−7.1
**16**	−8.3	−9.8	−4.9	−5.3	**60**	−8.0	−8.7	−6.4	−8.1	**101**	−8.1	−8.1	−5.5	−7.8
**17**	−7.9	−8.7	−5.6	−5.6	**61**	−8.2	−9.1	−6.2	−8.0	**102**	−7.9	−8.1	−5.9	−6.9
**18**	−8.3	−7.7	−5.3	−6.0	**62**	−7.9	−8.5	−5.3	−7.4	**103**	−7.9	−8.4	−5.2	−6.7
**19**	−9.2	−8.3	−5.8	−6.8	**63**	−8.3	−10.5	−6.2	−8.3	**104**	−8.4	−9.0	−5.2	−7.1
**20**	−9.2	−10.4	−6.7	−6.1	**64**	−8.9	−9.2	−5.7	−8.6	**105**	−8.0	−9.4	−5.7	−7.5
**21**	−9.2	−10.7	−6.7	−5.9	**65**	−8.0	−8.8	−5.8	−7.9	**106**	−7.8	−8.8	−5.6	−7.1
**22**	−9.0	−10.2	**−6.9**	−6.5	**66**	−8.8	−8.7	−6.0	−7.7	**miscellaneous**				
**23**	−8.7	−10.4	−5.8	−5.6	**67**	−8.3	−9.0	−5.4	−7.4	**107**	−8.1	−9.9	−5.2	−7.6
**24**	−9.0	−11.9	−5.6	−7.4	**guaianolides**					**108**	−7.8	−9.6	−5.8	−8.0
**25**	−8.7	−10.8	**−6.7**	−5.3	**68**	−8.1	−9.8	−5.5	−7.4	**109**	−7.3	−9.5	−5.0	−7.9
**26**	−8.9	−9.4	−5.9	−8.5	**69**	−7.8	−8.9	−5.8	−7.8	**110**	−6.5	−8.5	−3.8	−6.9
**27**	−8.6	−10.1	**−6.9**	−6.0	**70**	−8.0	−9.7	−5.8	−7.0	**111**	−8.8	−9.4	−5.3	−7.8
**28**	−9.4	−10.8	−6.5	−8.5	**xanthanolides**					**112**	−7.4	−7.9	−5.2	−7.0
**29**	−8.6	**−11.4**	−5.5	−7.7	**71**	−7.2	−9.4	−5.4	−6.8	**113**	−6.9	−8.8	−5.2	−6.7
**30**	−8.9	**−12.1**	−5.8	−7.0	**72**	−7.6	−8.5	−5.8	−7.2	**114**	−7.1	−9.1	−5.0	−7.1
**31**	−8.8	**−11.4**	−5.9	−8.5	**73**	−7.8	−8.9	−5.2	−7.4	**115**	−7.0	−9.6	−5.0	−6.7
**32**	−9.3	−10.2	−5.8	**−8.7**	**74**	−7.5	−7.9	−5.1	−7.1	**116**	−7.7	−8.1	−4.7	−6.9
**33**	−8.6	−9.8	−6.0	−7.6	**75**	**−10.6**	−11.4	−6.5	−0.5	**117**	−6.5	−8.5	−4.7	−6.7
**34**	−8.9	−11.2	−6.1	**−8.7**	**76**	**−10.6**	−9.4	−6.4	−3.3	**118**	−8.7	−9.2	−5.7	−7.6
**35**	−9.3	−10.6	−6.7	−7.0	**77**	−8.0	−9.5	−4.9	−7.3	**119**	−7.8	−8.4	−5.6	−7.6
**36**	−9.2	−9.5	−6.1	−7.2	**78**	−7.5	−8.6	−5.5	−7.4	**120**	−8.0	−9.0	−5.4	−7.2
**37**	−8.8	−9.5	−6.0	−8.4	**germacranolides**					**121**	−8.1	−7.5	−4.9	−7.1
**38**	−8.9	−10.0	−6.7	−6.6	**79**	−7.7	−8.6	−5.8	−7.4	**122**	−8.3	−9.2	−5.8	−7.7
**39**	−9.5	−9.4	−6.9	−6.1	**80**	−8.1	−9.3	−5.6	**−8.5**	**123**	−7.9	−8.6	−5.2	−7.7
**40**	−9.3	−10.4	−6.8	−5.9	**81**	−8.5	−9.1	−6.4	−6.7	**known inhibitors ^b^**				
**41**	−7.7	−7.0	−4.7	−6.6	**82**	−8.3	−9.7	−5.5	−7.8	I1	−10.2	−	−	−
**42**	−6.2	−6.3	−5.5	−7.0	**83**	−8.0	−10.6	−5.7	−8.3	I2	−	−9.6	−	−
**43**	−7.3	−7.1	−6.1	−5.3	**84**	−8.4	−8.9	−5.3	−8.2	I3	−	−	−8.0	−
**44**	−7.8	−6.8	−5.1	−5.6	**85**	−8.3	−8.8	−5.8	−8.1	I4	−	−	−	−8.2

*^a^* Vina scores (affinity values); *^b^* Reported inhibitors against each enzyme: I1 = DB07765; I2 = ZINC01690699; I3 = DDD64558; I4 = DDD66604.

**Table 3:** Mean values of docking energies (kcal/mol) and standard deviation for each skeletal type of sesquiterpenes with *Leishmania* enzyme targets.

Sesquiterpene−type skeletal	*Lm*PTR1		*Lm*CS		*Ld*NMT		*Lm*TryS	
	MDE *^a^*	%RSD *^b^*	MDE *^a^*	%RSD *^b^*	MDE *^a^*	%RSD *^b^*	MDE *^a^*	%RSD *^b^*
sesquiterpene coumarins	−8.8	11.4	−10.4	6.7	−6.0	11.0	−8.2	5.1
agarofurans	−8.5	9.0	−9.5	16.1	−5.9	11.5	−6.6	20.6
drimanes	−8.3	11.5	−9.6	14.3	−5.5	12.9	−7.8	7.0
pseudoguaianolides	−8.2	3.5	−9.1	6.7	−5.7	6.2	−7.8	5.9
guaianolides	−8.0	1.9	−9.5	5.2	−5.7	3.0	−7.4	5.4
xanthanolides	−8.4	16.9	−9.2	11.4	−5.6	10.5	−5.9	43.8
germacranolides	−8.1	3.9	−9.1	5.3	−5.7	5.8	−7.7	6.7
eudesmanolides	−8.0	2.6	−8.6	5.3	−5.4	5.6	−7.2	4.8
miscellaneous	−7.6	9.0	−8.9	7.5	−5.1	9.5	−7.3	6.2

*^a^* Mean values of docking energies (kcal/mol) for each skeletal type of sesquiterpenes; *^b^* Relative Standard Deviation (RSD) percentage of docking energy values for each skeletal type of sesquiterpenes.

**Figure 11:**
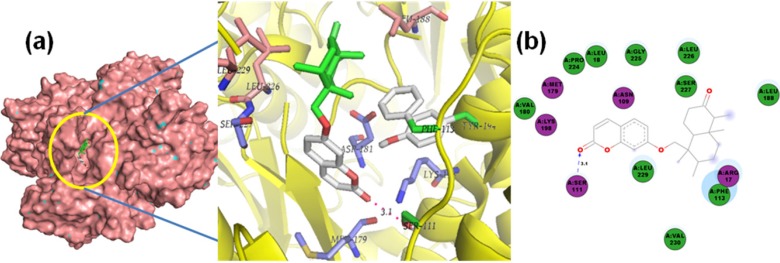
(**a**) Lowest-energy docked pose of sesquiterpene coumarin, szowitsiacoumarin A (**1**), with *Lm*PTR1; (**b**) 2D representation of protein ligand interactions of compound **1** with *Lm*PTR1.

On the other hand, the bicyclic sesquiterpene coumarins, szowitsiacoumarins A (**1**) and B (**2**), also exhibited good affinity with PTR1 (‒9.9 and ‒10.0 kcal/mol, respectively) very close to that of the control. The lowest-energy docked pose of compound **1** is shown in Figure 11. An identical *H*-bond interaction with Ser111 was revealed by the PTR1···**1** and PTR1···**2** complexes, which seems to be a key interaction for the complexes stabilization. Another polar interaction with Lys198 could also stabilize the complex as well as a receptor contact through Phe113 contributes to stabilization *via* the hydrophobic sesquiterpene centroid, which is located between three leucine (Leu226, Leu229, and Leu188) and one serine (Ser227) residues. These two compounds were reported by Iranshahi *et al.* [18]. However, their antiparasitic activity was not evaluated while compounds **4-5** were reported to be active against *L. major* promastigotes with IC_50_ values in the 13–17 µM range, but the PTR1 affinity values were not significant.

**Figure 12:**
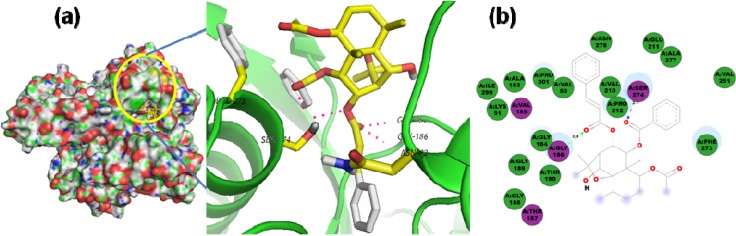
(**a**) Lowest-energy docked pose of agarofuran **30** with *Lm*CS; (**b**) 2D representation of protein ligand interactions of compound **30** with *Lm*CS.

Regarding docking with *Lm*CS, compounds **2**, **30**, and **75** exhibited the lowest docking energies (−11.8, −12.1, and ‒11.4 kcal/mol, respectively), possessing significant lower affinity values than that of control used in this study, ZINC01690699 (−9.6 kcal/mol). The agarofuran **30** was the most affine ligand for *Lm*Cs. The most-stable pose of **30** is shown in Figure 12. Two *H*-bond-type interactions are present in the CS···**30** complex when the ligand interacts with Gly186 and Ser274 through cinnamoyl and benzoyl oxygens at C-9 and C-8 carbons (2.4 and 2.1 Å, respectively). A structural requirement was observed on comparing the affinity values of isomers **29** and **30**. These compounds differ solely by the geometrical isomerism of the cinnamoyl double bond (*Z*-**29** and *E*-**30**). However, the docking energy of **30** was lower than that of **29** (−12.4 *vs.* ‒11.4 kcal/mol). The cynnamoyl aromatic ring is apparently necessary for a hydrophobic contact with Gly184 and Thr190 in **30**. The presence of the two cinnamoyl and benzoyl ester groups in **30** seems to be an essential requirement for the docking, since the best pose has these substitutions oriented towards the binding pocket, formed by Val50, Pro301, Phe273 and Ser274.

Interactions are represented as: van der Waals (green circle); polar (magenta circle); *H*-bond with amino acid side chains (blue dashed line); *H*-bond with amino acid backbone (green dashed line); pi interaction (orange line).

Agarofurans are considered as good therapies for Multidrug Resistant Drug (MDR)-agents [20,21]. The fact of 16 out of 35 agarofurans exhibited affinity values lower than −10 kcal/mol (with compound **30** as the strongest-docking sesquiterpene toward CS enzyme) indicates a rational starting point for the inhibition of the *de novo* route to produce cysteine in trypanosomatids.

Interactions are represented as: van der Waals (green circle); polar (magenta circle); *H*-bond with amino acid side chains (blue dashed line); *H*-bond with amino acid backbone (green dashed line); pi interaction (orange line).

**Figure 13:**
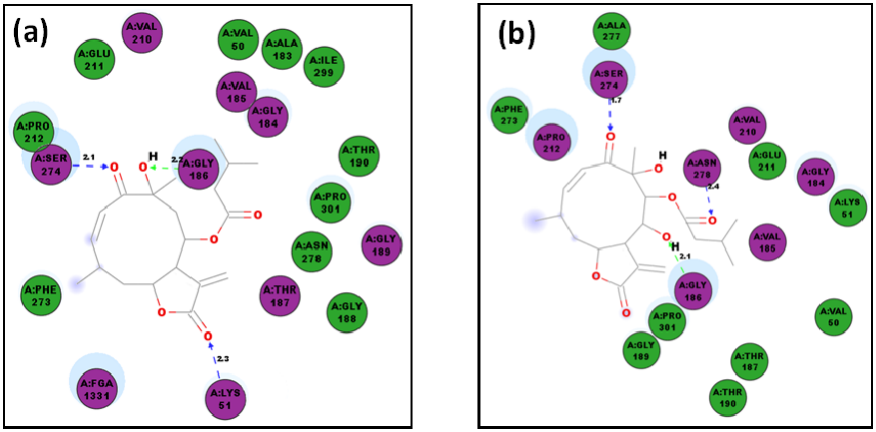
2D representation of protein ligand interactions with *Lm*CS of: (**a**) compound **83**; (**b**) compound **81**.

In addition, the germacranolide neurolenin A, **83**, also showed a better affinity value (−10.6 kcal/mol) than that of control for docking with *Lm*CS. Compounds **81** and **83** (which differ by the position of the isovaleoryl group attached at C8 and C9 carbons) exhibited affinities which were significantly different. On comparing the ligand interactions of these germacranolides, it is evidenced that the location of this ester group, although it is not determinant in the docking, is important since in the germacrene moiety in **83** allows suitable *H*-bond interactions with Gly186, Ser274 and Lys51 (*ca.* 2 Å) (Figure 13), while the lowest-energy pose of **81** is oriented in such a way that *H*-bond interactions with Gly186, Ser274 and Asn278 are presented. Seemingly, the hydrophobic proximity with Phe273, Asn278 and Glu211 contributes to the better stability of the *Lm*CS···**83** complex. Our results indicate that compounds **30** (in fact, several agarofurans) and **83** are promissory leads for further studies towards CS inhibition.

Interactions are represented as: van der Waals (green circle); polar (magenta circle); *H*-bond with amino acid side chains (blue dashed line); *H*-bond with amino acid backbone (green dashed line); pi interaction (orange line).

Germacranolide sesquiterpenes **81** and **83** also have an α-methylene γ-lactone moiety, but their respective lowest-energy poses are docked with different orientation. The lactone carbonyl of compound **83** exhibits a *H*-bond interaction (2.3 Å) with Lys51, which might promote a nucleophilic Michael addition. In contrast, the ligand interactions profile of compound **81** within the *Lm*CS active site does not exhibit a free –SH or –NH_2_-containing residue near to the ligand.

Interactions are represented as: van der Waals (green circle); polar (magenta circle); *H*-bond with amino acid side chains (blue dashed line); *H*-bond with amino acid backbone (green dashed line); pi interaction (orange line).

**Figure 14:**
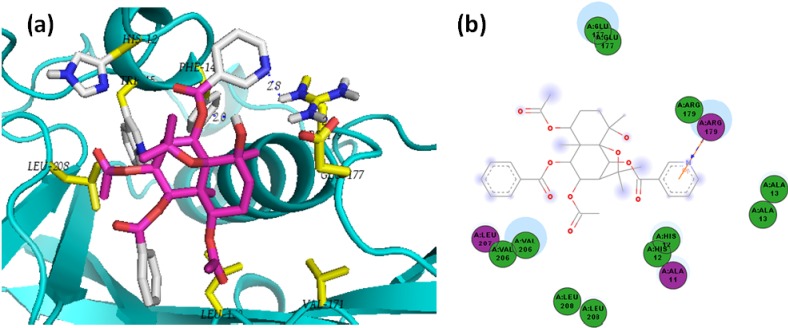
(**a**) Lowest-energy docked pose of agarofuran **22** with *Ld*NMT; (**b**) 2D representation of protein ligand interactions of compound **22** with *Ld*NMT.

Interactions are represented as: van der Waals (green circle); polar (magenta circle); *H*-bond with amino acid side chains (blue dashed line); *H*-bond with amino acid backbone (green dashed line); pi interaction (orange line).

Agarofurans **22**, **27**, and **39** exhibited the best docking energies (−6.9 kcal/mol) with *Ld*NMT, but the vina score of the control used, DDD64558, was substantially better (−8.0 kcal/mol). A *pi*-interaction was found between nicotinoyl moiety and Arg179 for all nicotinoyl-containing agarofurans evaluated in this study (Figure 14). The presence of this substituent was found to be critical to the docking of the agarofuran-related sesquiterpenes. Similarly, sesquiterpene coumarin **2**, exhibited almost identical affinity (−6.8 kcal/mol) with *Ld*NMT to that of **22**. However, after detailed analysis of the vina scores and binding modes of sesquiterpene···*Ld*NMT complexes, this kind of compounds could be considered as non-suitable ligands for NMT inhibition.

**Figure 15:**
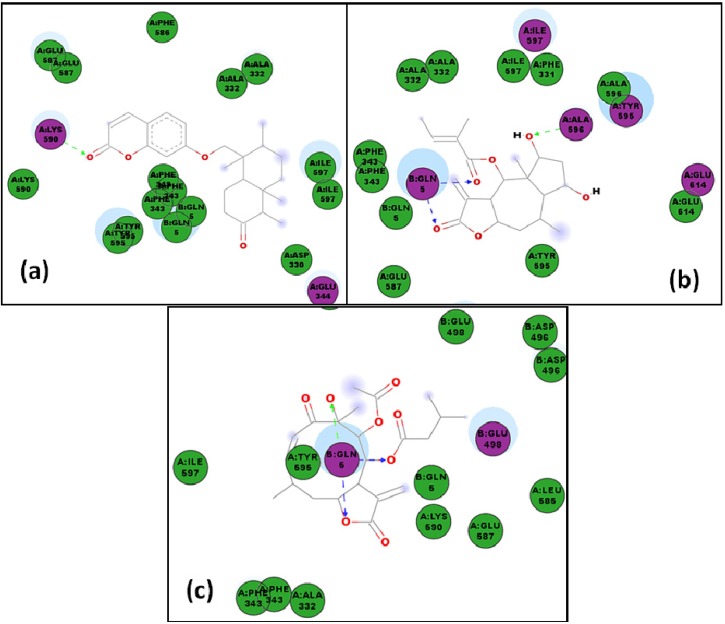
2D representation of protein ligand interactions with *Lm*TryS of: (**a**)compound **1**; (**b**) compound **64**; (**c**) compound **80**.

Sesquiterpene coumarins also exhibited good affinity values in the docking with *Lm*TryS, with compound **1** being the strongest-docking sesquiterpene coumarin (‒8.9 kcal/mol). Similarly, pseudoguaianolides **51** and **64** and germacranolides **80** and **89** exhibited good vina scores in the −8.5–−8.7 kcal/mol range. The complexes of all these compounds displayed lower docking energies than the control used, DDD66604, (−8.2 kca/mol). Figure 15 shows the 2D representation of protein ligand interactions of compounds **1**, **64** and **80**. The residue Gln5 was found to be a substantial polar contact in order to stabilize the complex for compounds **64** and **80**, while Lys590 resulted as the indispensable side chain donor contact for compound **1**. In addition, the lowest-energy pose of **80** has Lys590 very close to the α-methylene γ-lactone moiety.

Interactions are represented as: van der Waals (green circle); polar (magenta circle); *H*-bond with amino acid side chains (blue dashed line); *H*-bond with amino acid backbone (green dashed line); pi interaction (orange line).

**Figure 16:**
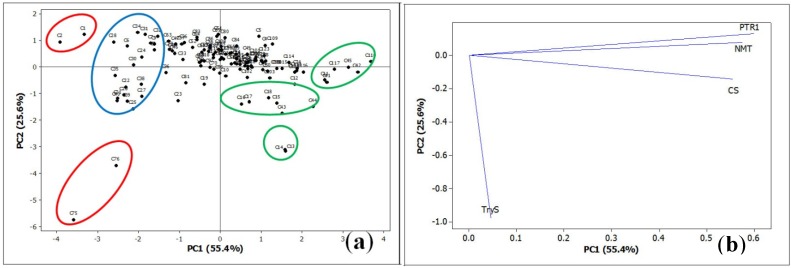
Principal Component Analysis for the docking energies of test compounds with *Lm*PTR1, *Ld*NMT, *Lm*CS, and *Lm*TryS. (**a**) Score plot; (**b**) Loading plot.

A Principal Component Analysis (PCA) was performed using the vina scores data of the test compounds with *Lm*PTR1, *Ld*NMT, *Lm*CS, and *Lm*TryS enzymes. The two first components of the PCA explain the 81% of the total variance. The resulting score plot (PC1 *vs.* PC2, Figure 16) revealed the clusters grouping the most affined ligands (red ellipses), on the one hand, and the ligands possessing the highest docking energies (green ellipses), on the other. The blue ellipse grouped the compounds with selectivity against one or two enzymes, but lowest docking values for the other enzymes. The resulting loading plot (PC1 *vs.* PC2) indicated that the docking values with *Lm*TryS have a substantially different behavior than other enzymes, whereas vina scores values with *Lm*PTR1, *Ld*NMT, and *Lm*CS are more highly correlated. Thus, compounds **75** and **76** are separated due to the higher docking values with *Lm*TryS but good affinity with the other enzymes, while sesquiterpene coumarins **1**–**2** are clustered since they exhibited overall stronger docking energies towards the *Leishmania* protein targets as mentioned above. These PCA results indicated the good *in silico* information-based discrimination as a reasonable tool for ligand design, structural optimization and combinatorial purposes. Additionally, the majority of sesquiterpene lactones is located at the center of the score plot, indicating that, although they may have a certain affinity level towards the test enzymes, in general their structural features not fit for competition of the active sites.

In order to observe the relationship between published IC_50_ values against *Leishmania* parasites and the docking energies for ligand design purposes, a correlation analysis was performed between Log(1/IC_50_) (IC_50_ in µM) *versus* vina scores (kcal/mol). However, due to the assay conditions-dependent variability of the data source and the limitation of the accuracy of docking calculations to predict *in vitro* activity, no significant correlation was then obtained (R^2^ < 20%). Such limitations in docking studies are well-known by the construction of the scoring functions through the postulation that solvation, entropy and electrostatics are largely applicable to several enzyme systems, adding compound absorption, plasma protein binding, tissue distribution, excretion and other biological events, which limit the prediction of biological activity. Nevertheless, there are several cases that distinguish between active and inactive compounds in docking studies and these procedures remain a powerful tool to facilitate the process of drug discovery [46,47].

Finally, in order to expand our study for identification of sesquiterpene-base leads, drug-like properties, such as partition coefficient (LogP), molar refractivity (MR), polar surface area (PSA), number of *H*-donors (*H*-D), *H*-acceptors (*H*-A) and rotatable bonds (RB), were calculated for the strongest-docking sesquiterpenes (Table 4). On applying the rule of five (RO5) and its variants [48], epimers **22**–**27** are violating more than one of RO5, such as MR, PSA, number of atoms and *H*-acceptors whereas agarofuran isomers **29**–**30**, agarofuran **24** and xanthanolides **75**–**76** exhibit an greater MR value to that of RO5 criteria. In contrast, sesquiterpene coumarins **1**–**2** have good drug-like properties, but the LogP values are near to the upper limit of lipophilicity. Within the strongest-docking sesquiterpenes, compounds **64**, **81** and **83** displayed the best lead-like properties, suggesting these compounds deserve additional studies for enzyme inhibition.

**Table 4:** Calculated druglikeness properties for strongest-docking sesquiterpenes.

Compound	LogP *^a^*	MR *^b^*	MW *^c^*	MF *^d^*	PSA *^e^*	*H*-D *^f^*	*H*-A *^g^*	RB *^h^*
1	5.50	110.2	382.5	C_24_H_30_O_4_	52.6	0	4	3
2	4.78	112.2	382.5	C_24_H_30_O_4_	55.8	1	4	3
22	2.03	149.7	595.6	C_32_H_37_NO_10_	147.6	1	11	10
24	3.48	152.7	578.6	C_33_H_38_O_9_	128.6	2	9	9
27	2.03	149.7	595.6	C_32_H_37_NO_10_	147.6	1	11	10
29	4.61	151.5	562.7	C_33_H_38_O_8_	108.4	1	8	9
30	4.61	151.5	562.7	C_33_H_38_O_8_	108.4	1	8	9
31	3.22	120.5	442.5	C_26_H_34_O_6_	82.1	1	6	6
39	5.06	168.7	641.7	C_37_H_39_NO_9_	127.3	0	10	11
51	1.08	70.6	262.3	C_15_H_18_O_4_	63.6	1	4	0
63	1.70	95.6	364.4	C_20_H_28_O_6_	93.1	2	6	3
64	1.70	95.6	364.4	C_20_H_28_O_6_	93.1	2	6	3
75	1.74	139.4	508.6	C_30_H_36_O_7_	107.0	1	7	3
76	0.82	137.9	524.6	C_30_H_36_O_8_	119.5	1	8	3
81	1.22	99.0	380.4	C_20_H_28_O_7_	110.1	2	7	4
83	1.86	97.6	364.4	C_20_H_28_O_6_	89.9	1	6	4

*^a^* partition coefficient; *^b^* molar refractivity (cm^3^·mol^−1^); *^c^* molecular weight (g/mol); *^d^* molecular formula; *^e^* polar surface area (Å^2^); *^f^* number of *H*-donor elements; *^g^* number of *H*-acceptor elements; *^h^* number of rotatable bonds.

## 3. Experimental Section

### 3.1. Protein Preparation

The X-ray crystallographic structure of *Lm*PTR1, *Ld*NMT, *Lm*CS, and *Lm*TryS proteins were obtained from the Protein Data Bank at a resolution of <2.5Å. Water molecules, ligands and other hetero atoms were removed from the protein molecule along with the chains excepting chain A. Addition of hydrogen atoms to the protein was performed. Energy minimization was performed with the CHARMm force field by using conjugate gradient method with an RMS gradient of 0.01 kcal/Å mol on Discovery Studio 2.5 software [49].

### 3.2. Preparation of the Ligands

Structural information of 123 test sesquiterpenoids was acquired from a literature survey on natural sesquiterpenes with antileishmanial activity report (see supplementary material for the IUPAC and common names). A Monte-Carlo randomized conformational search, without any geometrical restrictions, was made using the Merck Molecular Force Field (MMFF94) included in the SPARTAN software [50] with a limit of 500 conformers. Energetically lowest stable conformers within a 6 kcal/mol energy range were geometrically optimized using the semi-empirical AM1 parametrization implemented in the software package. The minimized protein and ligands were saved in PDB format for further docking analysis.

### 3.3. Docking Analyses

Docking experiments were performed using the Autodock/Vina (1.1.2) package under Linux in a Python 2.7 environment, using the AMBER force field [51]. The protein and ligand molecules were prepared as described above. The docking experiment on test enzymes was carried out between the energy-minimized ligand into the active site through a cube at the geometrical center of the native ligand present in the evaluated PDB structure, with the dimensions 24 × 24 × 24Å, covering the ligand binding site with a grid point spacing of 0.375Å. The docking poses are ranked according to their docking scores (as free energy of binding) and both the ranked list of docked ligands and their corresponding binding poses were exported as a CSV file for further analysis. The docked structures were viewed using PyMOL (1.3r2) [51].

### 3.4. Ligand Interactions

Resulting docking information-containing files (in .pdbqt format) were imported into the Accelyrs Discovery Studio 2.5 software package. Once the docked enzyme-ligand complex was imported, an analysis of the binding site was performed in order to create a ligand interaction 2D diagram.

### 3.5. Drug-Like Properties Calculations

Drug-like properties of each sesquiterpene were separately calculated using the Molecular Topology and ChemProPro plugins available into the ChemBio3D^®^ Ultra 11.0 software package (Cambridge Soft Corporation, Cambridge, MA, USA) starting from the 3D structure.

### 3.6. Statistics

Regression and PCA analyses were carried out using the algorithms included into the software R (3.0.1 version) [52].

## 4. Conclusions

A plethora of antileishmanial naturally-occurring compounds active against promastigotes and amastigotes have been identified and evaluated, but their mode of action has not been elucidated. A great number of sesquiterpenes remains in the available literature considered as good antileishmanial leads, e.g., sesquiterpene lactones. However, the lack of molecular studies is a barrier in the route for improving the sesquiterpene-based drug design. In the present *in silico* study, we have identified molecular details of the binding modes of a set of antileishmanial sesquiterpenes against four drug-enzyme targets (*Lm*PTR1, *Ld*NMT, *Lm*CS, and *Lm*TryS). Through molecular docking it was found that two sesquiterpene coumarins are good ligand candidates for the PTR1 and TryS inhibition purposes as well as some xanthanolides exhibited better affinity towards PTR1 and CS binding. The ligand interactions allowed establishing the critical contacts between the each ligand and the active site of the enzymes. The present study is an excellent starting point for future studies of structural optimization of these compounds, whose observed details at the molecular level allowing opening a working route towards developing therapeutic sesquiterpenes-based prototypes against *Leishmania*.
